# Nursing Epidemiological Approach of Hypertension Management in a Public Health Service from the Northern Region of Portugal

**DOI:** 10.3390/healthcare9010059

**Published:** 2021-01-08

**Authors:** Pedro Melo, Dário Miranda, Sandra Santos, Sérgio Sousa, Teresa Cardoso, Alexandra Pereira

**Affiliations:** 1 Universidade Católica Portuguesa, Centre for Interdisciplinary Research in Health, 4169-005 Porto, Portugal; 2 Universidade Católica Portuguesa, Institute of Health Sciences/School of Nursing (Porto), 4169-005 Porto, Portugal; enf.dario.miranda@gmail.com (D.M.); sanasantos@gmail.com (S.S.); 3 Public Health Unit, Local Health Unit of Matosinhos, 4450-021 Matosinhos, Portugal; sergio.sousa@ulsm.min-saude.pt (S.S.); teresa.cardoso@uslm.min-saude.pt (T.C.); 4 Nursing Information System Department, Portugal Northern Health Administration, 4000-477 Porto, Portugal; alemnap@gmail.com

**Keywords:** public health nursing, epidemiological surveillance, nursing diagnosis, arterial hypertension

## Abstract

Background: Epidemiological surveillance of a nursing diagnosis is an approach anchored in a post-modern epidemiology focused on a person’s health disease responses. Regarding public health priorities, the population where our study occurred had as a priority problem arterial hypertension. Related to this chronic disease, nursing diagnoses about health disease responses in primary healthcare has, as a major focus, Therapeutic Regimen Management. Our aim was to study the nursing diagnosis in this issue from an epidemiological approach. Methods: A descriptive study from an epidemiological approach was developed, analyzing nursing diagnoses in hypertensive patients. Results: We found 17.7% of undiagnosed patients and better diagnoses in patients with complications than in those without complications. Conclusions: Nursing records need to be improved in order to promote more robust studies in the post-modern epidemiology for the future.

## 1. Introduction

The first references to epidemiology date back to V BC and lasted until 1830. Perhaps the work of Hippocrates “Air, Waters and Places” is one of the first references to an epidemiological analysis of infectious diseases (even without understanding them from a microbiological point of view) and its relationship with the environment [1].

Epidemiology has been developing its focus as a methodology, expanding the analyses of public phenomena from disease distribution, starting with infectious diseases (classical epidemiology) [2] to an eco-epidemiology focus in societal and molecular level pathways [3] to interactions between the individual and environment [4] and a recent focus on health disease phenomena, with a post-modern epidemiology based on Public Health Nursing science [5]. This means that this post-modern epidemiology assesses, from an epidemiological point of view, the intentional processes (based on knowledge, beliefs or motivation); the unintentional processes (e.g., the physiological) and the interaction processes with the environment (based on the nursing science meta-paradigmatic concept of a person) [6].

**Figure 1:**
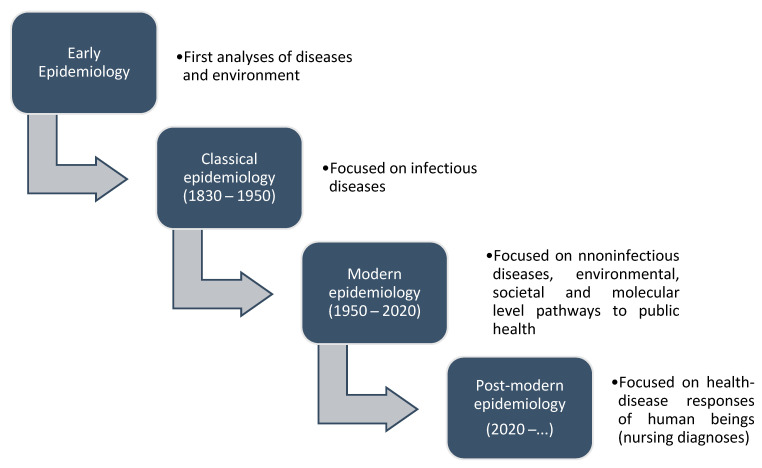
Development of epidemiology (adapted from Melo (2020) [5]).

The evolution of epidemiology is presented in Figure 1.

In Portugal, there are different Information Technology (IT) systems that contribute to epidemiological surveillance. In primary healthcare, nurses make their care records in an IT software called *S-Clínico* that allows the record of diagnosis activity, diagnoses and interventions based on the International Classification for Nursing Practice—ICNP [7]. Since 2016, all the parametrization of *S-Clínico* has been standardized throughout the country, with the same codes for diagnoses and interventions, preventing free documentation by nurses with different terms or codes for the same diagnoses and interventions.

Thus, nurses who are specialists in Community Health and Public Health Nursing currently have legal standing to lead the epidemiological surveillance of their diagnoses [8]. Moreover, they have a robust IT documentation of all nurses in primary healthcare and are allocated to public health units in a regulated manner [9], being this a perfect context to develop the epidemiological surveillance of nursing diagnoses.

All of these conditions are great opportunities to develop the post-modern epidemiology defended by Melo [5]. However, a recent study developed in this problem identified that primary healthcare structures in Portugal have a low level of empowerment to develop the epidemiological surveillance of nursing diagnoses, despite the good environmental and organizational conditions existing nowadays in the country [10].

As epidemiological surveillance of nursing diagnoses is not yet a reality, the understanding of its development from a medical perspective can help identify the actual major priorities in public health and introduce through this new approach in a most understandable way the importance of nursing diagnoses to others that are not nurses.

In Portugal, there is an IT software that allows the data analyses of mandatory reported diseases, called SINAVE (National System of Epidemiological Surveillance). This software allows producing important public health data related to diseases prevalence, incidence and its relation with other public health phenomena, such as environmental issues, for example.

Each Public Health Unit produces annually a Local Health Diagnosis from data extracted from SINAVE and other data produced locally by local IT software. This Local Health Diagnosis guides the development of the Local Health Plan annually, which responds to the health needs of that population.

From the analyses of Local Health Diagnosis from the Public Health Unit that was the epicenter of this study, the priorities of intervention based on major local diseases were focused in cardiovascular diseases, mainly arterial hypertension.

The medical reports on arterial hypertension diagnoses evidenced two important codifications of these diagnoses based on the International Classification of Primary Care (ICPC-2) [11]:

(1) K86—referring to the medical diagnosis of arterial hypertension without complications.
(2) K87—referring to the medical diagnosis of arterial hypertension with complications.

Both doctors and nurses in Portuguese primary healthcare units document their care in the IT *S-Clínico* but each one with the respective profiles of the doctor or nurse. These diagnoses, related to hypertension described above and read by SINAVE, are documented in the IT software *S-Clínico* in the doctor profile of *S-Clínico*.

**Figure 2:**
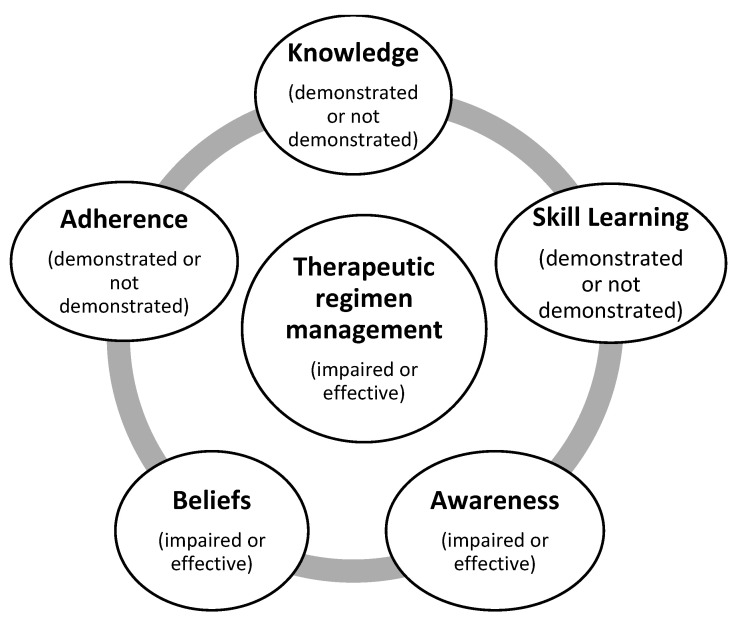
Nursing Diagnostic criteria for therapeutic management in chronic disease patients [7,12].

Nurses, from these reports, identify the chronic patients with arterial hypertension, activate their consultation process and start the Nursing Diagnosis regarding the chronic disease. One of the major Nursing Diagnoses in this problem of chronic diseases is related to “Therapeutic Regimen Management” [12,13]. This nursing focus can be diagnosed as impaired or effective, based on the clinical diagnostic criteria related with knowledge about the therapeutic triangle; food, medication and physical activity; blood pressure self-monitoring skill learning; awareness about the chronic condition and lifestyle; beliefs about the disease and therapeutic management and adherence to therapeutic management, as presented in Figure 2.

In these circumstances, we have potentially a clinical relationship between the nursing diagnosis related to the therapeutic management of arterial hypertension (codified according to the ICNP) and medical diagnosis of arterial hypertension (with complications (K87) or without complications (K86), codified according to the ICPC-2.

The interrelation between these different health professionals, responding to local public health needs (although currently based more on disease than in health disease responses) demands an empowerment of primary healthcare communities to promote this new epidemiological approach. This process should be based on community partnerships that can be intentionally promoted by public health nurses [14] in a process of community empowerment to promote the epidemiological surveillance of nursing diagnoses [10].

In this context, the aims of our study are:

a) to identify the distribution of nursing diagnoses in Therapeutic Regimen Management related to the codifications of medical diagnoses associated with arterial hypertension and
b) to identify the documentation rates for nursing diagnoses related to the Therapeutic Regimen Management in hypertensive patients.

## 2. Materials and Methods

The study had an epidemiological approach, with a quantitative methodology, considering a descriptive analysis of the data record on *S-Clínico* between 1 January 2019 and 31 December 2019. 

It was asked of the Department of Data Management of the primary healthcare organization where the study was developed to extract the data recorded on *S-Clínico* considering the follow criteria:

- Persons with valid registration in the health centers of the primary health care organization (some of registration records were excluded, because they were repeated, referred to persons that already died or were sporadic registrations of persons who did not live in the area covered by the health center, but they occasionally needed care and needed to be registered to access it), considering the inclusion criteria:
  ○ activation of the program of hypertension follow-up,
  ○ medical diagnosis of hypertension with complications (K87),
  ○ medical diagnosis of hypertension without complications (K86),
  ○ activation of the focus Therapeutic Regimen Management (without the nursing diagnosis complete),
  ○ complete diagnosis of the impaired or effective Therapeutic Regimen Management (with the standard national codification) and
  ○ complete diagnosis of impaired or effective Therapeutic Regimen Management (instead of the standard national codification).

The data were treated using descriptive statistics, with the Microsoft Excel 2020 program, and it identified the:

- relation between nursing diagnosis activity and medical diagnosis of arterial hypertension in general, arterial hypertension with complications and arterial hypertension without complications (comparing each diagnoses prevalence). These calculations were made by extracting the data from the IT *S-Clínico* and calculating the prevalence coefficient for each diagnosis, considering the codes K86 and K87 for the medical diagnosis and the judgment impaired or effective to the nursing focus Therapeutic Management (as described in the INCP and standardized in the national IT *S-Clínico*).
- relative frequencies of Nursing Diagnoses identified in Therapeutic Regimen Management (judgment impaired and effective) and others relative to judgements previous to the 2016 standardization of the terms impaired and effective.
- relative frequencies of undiagnosed Therapeutic Regimen Management focus (the focus activated but without any record of positive or negative judgement).

In this phase, considering the initial stage of the observatory of nursing diagnoses, it did not develop a deeper analysis; that it, is intended to be promoted when the nursing records can effectively be improved.

The study was submitted and approved by the institutional ethical committee with the ethical approval code N 55/CE/JAS from 13/07/2018.

## 3. Results

We found a record of 134,536 registered users, of which 56% were women and 44% men. The distribution of the prevalence of medical and nursing diagnoses are presented in Table 1.

The prevalence of general hypertension in the population in the study was 29% (25% without complications and 3.8% with complications). From the total of patients with arterial hypertension, 65% were males and 35% females. We did not analyze more deeply this data because that was not the aim of our study.

**Figure 3:**
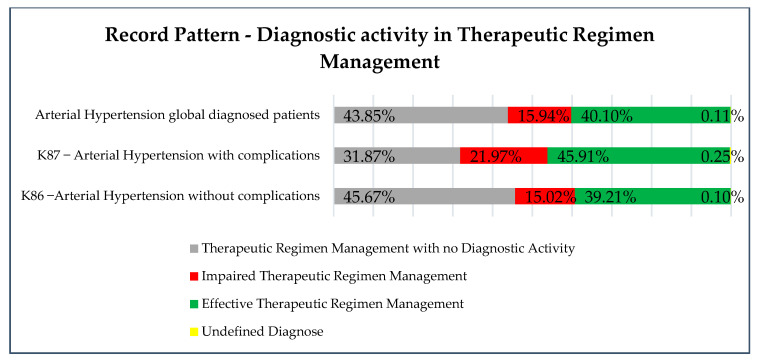
Distribution of nursing diagnosis activity in Therapeutic Regimen Management.

Responding to the aim of identifying the distribution of nursing diagnoses in Therapeutic Regimen Management related to the codifications of medical diagnoses associated with arterial hypertension, the results are shown in Figure 3.

Globally, considering the 39,059 hypertension patients as described in Table 1 (without analyzing the complications or non-complications), 43.85% (17,127 patients out of 39,059) had activation of the focus “Therapeutic Regimen Management” but without any kind of judgment (e.g., impaired or effective) that would define the nursing diagnoses. This means nurses activated the program of hypertension follow-up and clicked on the focus code in the IT *S-Clínico* in the patient nursing process. However, if this happened before the standard national codification that demands the attribution of the judgment of impaired or effective (concerning diagnosis criteria), the diagnosis was not record in *S-Clínico*. The patients (56.04%; 21,888 out of 39,059), meanwhile, had a complete diagnosis: 15.94% (6226 out of 21,888) a diagnosis of impaired Therapeutic Regimen Management and 40.10% (15,662 out of 21,888) an effective Therapeutic Regimen Management. Some patients (0.11%; 44 out of 39,059) had an undefined diagnosis (the system identified it as an unspecified error).

**Figure 4:**
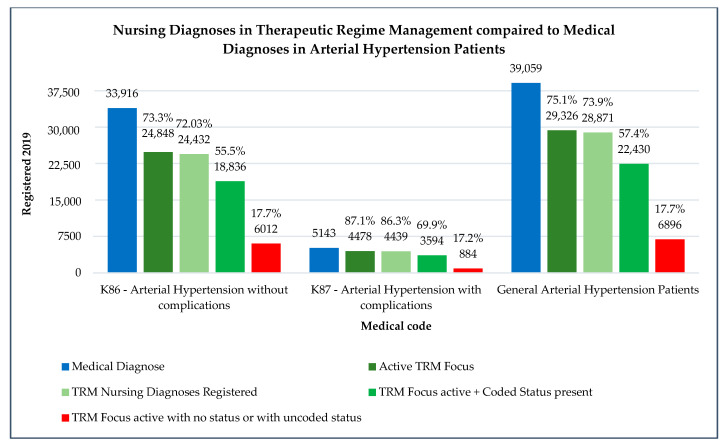
Nursing diagnoses in Therapeutic Regimen Management compared with medical diagnoses in arterial hypertension patients.

Concerning the aim of identifying the documentation rates for nursing diagnoses related to Therapeutic Regimen Management in hypertensive patients, the results are presented in Figure 4.

Globally, patients with arterial hypertension diagnoses have 73.3% Therapeutic Regimen Management activation by nurses. When referred to nursing diagnoses, it recorded 72% of hypertension patients with complete nursing diagnoses. Some patients (55.5%) had nursing diagnoses according to the national documentation standards defined in 2016, and 17.7% had no complete diagnosis (just focus activation without a complete assessment and clinical judgement or with uncoded status (free writing judgments uncoded that do not allow a diagnosis interpretation in an epidemiological approach)).

When comparing arterial hypertension patients with complications to those without complications, nursing diagnoses are more expressed in the first ones, with 87.1% Therapeutic Regimen Management activation by nurses against 75.1% in the second one. The other data (related with complete diagnoses and coded diagnoses) are also higher represented (86.3% and 69.9% against 75.1% and 73.9%, respectively). The rates of patients with uncoded diagnoses or no diagnoses are similar in both patients (17.2% in K87 patients and 17.7% in K86 patients).

The population of hypertensive patients with complications is much lower than the patients with arterial hypertension without complications (5143 and 39,059, respectively).

## 4. Discussion

Our results evidence that a nursing diagnosis is needed to increase the quality in order to promote robust epidemiological surveillance.

The rate of uncoded diagnoses needs to decrease to lower levels, even in this reality, considering of the totality of hypertensive patients, 17.7% can be potentially easily improved. The low community empowerment levels shown by our previous study in this issue [10] reveal the importance of empowering intervention by community and public health nurses to primary healthcare nurses as a whole regarding the epidemiological surveillance of nursing diagnoses.

Patients with complications, although in lower number, have better rates of a nursing diagnosis, regarding to the Therapeutic Regimen Management. This can be related with the fact that these patients have more nursing consultations with family health nurses. The higher prevalence of effective Therapeutic Regimen Management compared with the impaired one is also a good indicator of the state of control of disease for hypertensive patients. In this matter, the patients with complications have a higher prevalence of effective Therapeutic Regimen Management when compared with patients without complications. Maybe this orientates to the necessity of increase nursing consultations in these patients in order to avoid the development of complications.

Arterial hypertension is an important issue with impact in the public health perspective in the population studied. However, nurses still do not evidence their contribution in an objective path (revealed by their records and the epidemiological approach of it). This would consolidate the data analyses relating nursing diagnoses with public health determinants in the population, with the potential recognition of the added value provided by nurses to the population.

Although this new epidemiological approach focused on nursing diagnoses finds a perfect context in Portugal to be developed with the perspective defended by Melo [5,14] and anchored in Nursing Science in its way of looking at the person in the intentional, unintentional and environmental interaction processes [6], it is still making its first steps.

With our research were created the first nursing diagnosis observatories, based in public health units, involving all nurses and other organizations with intentional community partnerships related with the epidemiological approach of nursing diagnoses [14].

Other studies must be developed regarding comparative and correlational studies, but for now, this descriptive study and, at the same time, mixed approach studies related to community empowerment being developed, can definitely improve the conditions to promote more robust epidemiological studies in the future. These conditions are related to community-based interventions that promote increased levels of empowerment regarding:

- the development of organizational structures (such as nursing diagnostics observatories),
- the promotion of partnerships with structures that improve the epidemiological surveillance of nursing diagnoses (such as the Portuguese Ministry of Health itself),
- the improvement of leadership, concerning the knowledge and attitudes related to the IT documentation itself and epidemiological surveillance and
- the improvement of communication processes in the nurse community.

The use of a community-oriented nursing reference as a client, such as the one we are using in our investigation, the Community Assessment, Intervention and Empowerment Model (MAIEC) [5,14], is also an improvement factor to promote the epidemiological surveillance of diagnoses of nursing.

The existence of major interventions is related to Therapeutic Regimen Management [7] and related to the different diagnosis dimensions presented in the Introduction of this manuscript (Figure 2), such as:

- teaching or informing (related to knowledge),
- instructing (regarding skill learning (e.g., the self-monitoring of glycemic values),
- promoting awareness,
- promoting facilitating beliefs and
- promoting adherence to a therapeutic regimen.

All these interventions, associated in the case of hypertension to food, exercise and medication, and its appropriate record in IT by nurses that provide it, can assure that the epidemiological studies can find the irreplaceable contribution of nurses to the control of arterial hypertension with the support of specialist nurses in community health and public health nursing who promote, through epidemiological surveillance, the value of the data that all nurses produce can be found. Moreover, behind robust data is robust care and the population’s access to quality nursing care—in this case, in the context of arterial hypertension.

Despite the evidence of needed improvements in nursing records, concerning arterial hypertension, we found good results, with rates of global nursing diagnoses above 70%. This means that nurses are making efforts to contribute to the assessment of the population state of health from this scientific perspective. With the organizational context promoter of improvements and the increase of nursing community empowerment, the epidemiological surveillance of nursing diagnoses can be a reality really soon.

## Figures and Tables

**Table 1:** Distribution and prevalence of medical diagnoses in hypertension patients.

Medical Diagnose	*n*	Prevalence
Arterial Hypertension patients	39,059	29%
K86—Arterial Hypertension without complications	33,916	25%
K87—Arterial Hypertension with complications	5143	3.8%

## Data Availability

Not applicable.
